# Exploring Trends in Environmental, Social, and Governance Themes and Their Sentimental Value Over Time

**DOI:** 10.3389/fpsyg.2022.890435

**Published:** 2022-06-28

**Authors:** Joonbeom Park, Woojoo Choi, Sang-Uk Jung

**Affiliations:** ^1^Graduate School of Information, Yonsei University, Seoul, South Korea; ^2^Graduate Business School, Hankuk University of Foreign Studies, Seoul, South Korea

**Keywords:** topic modeling, Latent Dirichlet Allocation (LDA), dynamic topic model (DTM), sentiment analysis, ESG, Twitter data, natural language processing (NLP)

## Abstract

Environmental, social, and governance (ESG) is an indicator that measures a company’s non-financial performance. Many firms have recently emphasized the importance of ESG. Ascertaining what topics are being discussed around ESG and how they change over time will contribute significantly to gaining insight into ESG. Using 73,397,870 text data scraped and refined from publicly available Twitter data, this study applied Latent Dirichlet Allocation (LDA) and the dynamic topic model (DTM) to ascertain the hidden structure of the ESG-related document collection and the topics being discussed. The study further conducts a sentiment analysis to examine the sentiment of the general public regarding ESG. Topic modeling shows that various topics regarding ESG are being discussed and evolve over time. Sentiment analysis shows that many people have neutral or positive sentiments toward ESG-related issues. This study contributes to exploring insights into ESG among the public and understanding public reactions toward ESG. We further conclude the study with a discussion of managerial implications and potential future research.

## Introduction

There is no management concept that has been receiving as much attention as environmental, social, and governance (ESG) recently. To be ESG-compliant (an extension of corporate social responsibility and sustainability) used to merely mean being a “good company.” Recently, however, ESG has come to mean a “company strong in crisis.” In other words, how well a company can respond to and continuously manage risks, such as “environmental, social, and governance” risks, is becoming crucial in the face of increasing uncertainty in the corporate environment (Jo and Na, 2012). Particularly, many companies have been affected negatively by the recent COVID-19 pandemic. Interest in ESG has further sparked in terms of crisis management. For example, Díaz et al. (2021) show a change in the stock price gap between the top 25 and bottom 25 companies of the S&P 500 since the outbreak of the COVID-19, proving that companies with higher ESG ratings can mitigate the crisis.

Along with financial performance, the ESG index has become a significant indicating factor of a company’s non-financial performance. Moreover, investments based on it have recently become the “new normal.” According to Broadridge Data and Analytics (2021), ESG investment has increased to approximately seven trillion dollars; it is predicted that by 2030, it could increase to 30 trillion dollars. This expansion of ESG investment stems from the fact that the ESG concept emerges from the needs of investors who want to adequately evaluate the value of a company. Corporate social responsibility (CSR) is considered the incidental activity of a company to enhance corporate image and reputation. Accordingly, there are limitations in linking it with corporate management performance. In contrast, ESG pursues corporate sustainability based on environmental, societal, and governance themes. Moreover, the evaluation of corporate non-financial performance and investment are the most important factors. Rather than examining these changes, academia often considers ESG similar to the CSR concept and applies previous research perspectives and methods as they are. Therefore, there are few empirical studies on ESG. Moreover, most existing research focuses on the relationship between ESG and corporate performance or ESG assessment and evaluation.

Our study focuses on the ESG theme and the changes in its trends. We derive the most discussed topics about ESG and explore how society interprets and accepts ESG, and how society’s understanding and interest in ESG change over time. We achieve this by scraping vast amounts of text data from Twitter using keywords, ESG, and #ESG, and analyzing the unstructured text content using machine learning techniques (ML). Twitter was used as a data source because it allows us to obtain a large amount of textual data of public opinion on ESG in real time (Gaytan Camarillo et al., 2021). Although information about ESG is abundant in corporate official documents and other social media services, we chose “public” Twitter as our data source because no other social media channel makes sharing information as easy. In other words, Twitter is suitable for our study, which aims to analyze ESG trends, as it allows for reading real-time trends through powerful information sharing (Vargas et al., 2021). The types of data also vary, from office domains such as businesses and governments to personal messages, which helps generalize the results of the analysis.

This study analyzes ESG-related big data in Twitter texts and classifies ESG-related Twitter conversations into several topics. Additionally, we examine the changes and trends in these topics over time. We further conduct sentiment analysis to examine society’s overall sentiments toward ESG.

This study differs from most existing studies that consider ESG a performance or evaluation target. We focus more on the concept of ESG. We further examine what the general public is saying and communicating about ESG, its sentiment toward ESG, and how these conversations and emotions change over time.

This study can be replicated by scraping and analyzing tweet data in real-time. This can help managers understand public sentiment, gain insights into ESG, and establish management strategies. Analyzing the discussion of ESG in tweets worldwide also provides a global perspective.

Our research questions are

(1) What is the latent topic structure of ESG?
(2) How have the structures of ESG-related topics changed over time?
(3) What are the sentiments of ESG-related discussions?

This paper proceeds as follows. See section “Literature Review” briefly reviews related research. See section “Data and Methodology” presents the data and the model. See section “Empirical Results” discusses the findings and implications. See section “Discussion” further presents the conclusions and future research directions.

## Literature Review

### Environmental, Social, and Governance

The ESG concept refers to environmental, social, and governance structures, which are non-financial factors of a company, and is among the main indicators for evaluating a company’s value. The UN emphasizes that a systematic response to ESG is essential for companies to achieve sustainable growth in future and, accordingly, devised the ESG concept in the UN Global Compact (2004), to highlight non-financial issues that may or may not significantly affect a company’s investment value.

To understand ESG better, there is a need to understand the concept of corporate social responsibility (CSR), which is the precursor to ESG. Since Friedman (1970) seminal work, the discussion on the role of business in society has continued and expanded for more than 50 years. Accordingly, CSR has become an important element in corporate management and has been actively studied academics (Lee, 2008; Wang et al., 2016). However, it is becoming increasingly difficult to objectively define CSR and measure its performance as society’s complexity increases and the scope of CSR expands to shareholders, society, customers, the economy, and the environment (Khojastehpour and Shams, 2020).

The ESG concept has been widely used as an alternative to CSR in the media, and in many studies; ESG and CSR are often defined as the same concept. However, ESG is more detailed than CSR in terms of definition and evaluation. While CSR aims to hold companies accountable, ESG defines the social role of companies in three domains: environmental, social, and governance (Dahlsrud, 2008). Moreover, CSR considers all stakeholders in a company while ESG focuses on ESG investors.

The idea of ESG began with investors’ concerns and dissatisfaction with traditional CSR. Although companies’ CSR activities may have helped enhance corporate image and reputation, investors were skeptical about whether they were effective and strategic in enhancing long-term corporate value. There were also complaints about companies’ disclosure of CSR information. Corporate CSR reports had promotions and boasted about CSR activities but had little connection with corporate performance. Moreover, traditional financial statements could not report ESG performance adequately. Factors such as corporate carbon emissions, corporate governance, and stakeholder management were not disclosed in financial statements (Amel-Zadeh and Serafeim, 2018).

Various disclosure standards and frameworks of ESG information, including the Sustainability Accounting Standard Board (SASB) and Taskforce on Climate Related Financial Disclosure (TCFD), have been suggested. The SASB was established to connect investors and companies in terms of ESG performance. The board divided companies into 77 sectors and selected factors that have high investor interest and financial relevance among the ESG factors that affect the company’s financial performance, production, and operating activities in each sector (Busco et al., 2020). The TCFD was established by the G20 finance ministers and central bank heads of each country to establish climate change-related disclosure guidelines for companies. Companies must explain how climate change-related risks and opportunities are to be managed and how they relate to financial statements (Nelson, 2018).

The corporate standards of institutions such as “MSCI” and “Sustainalytics,” which existed before ESG, were developed in line with the new ESG concept (Escrig-Olmedo et al., 2019). Moreover, companies increasingly made efforts to convert ESG standards into corporate profits by applying ESG standards to all areas of their business. Further, many investors created funds to invest in companies with high ESG performance based on these ESG standards.

Based on this ESG concept, ESG evaluation indexes of various ESG evaluation agencies such as “MSCI” and “Sustainalytics” were created and developed (Escrig-Olmedo et al., 2019). Additionally, companies converted ESG indicators, which many investors use to create funds that invest in companies with high ESG performance, into actual business profits. In 2020, global ESG investments more than doubled to $371 billion (Broadridge Data and Analytics, 2021), demonstrating just how significant the effect of ESG was on the business.

Additionally, concerns about the effects of environmental and climate change have raised the public demand for corporate accountability and transparency. Increasing public engagement, especially following the spread of social media, increases public pressure. Moreover, companies make ESG a top corporate agenda.

From this perspective, it is meaningful to examine the concept of ESG and examine the flow of change and the general public’s sentiment toward ESG.

### Research on Environmental, Social, and Governance in Business

Environmental, social, and governance-related research in the business area mainly focuses on evaluating ESG and analyzing the relationship between ESG and corporate performance. ESG assessment can be considered a continuum as it is a prerequisite for corporate performance analysis.

Research on the relationship between ESG and corporate financial performance has been ongoing for more than 40 years. Whether a company with high ESG can generate good financial performance has been a topic of interest for many scholars. Friede et al. (2015) conducted a meta-analysis of over 2,000 related papers on the relationship between ESG and corporate financial performance (CFP). ESG and CFP are classified as positive, negative, and neutral; the negative relationship accounts for only 10%. Similarly, most CSR research shows that CSR has a positive effect on corporate performance (Becker-Olsen et al., 2006).

There are several explanations for the positive relationship between ESG and CFP. Bénabou and Tirole (2010) analyzed three motivations for corporate ESG initiatives: adopting a long-term view of the company, delegating pro-social action on behalf of stakeholders, and insider-led corporate philanthropy. Thus, corporate performance can be created in the process of avoiding short-sighted judgments and increasing shareholder value from a long-term perspective. Baron (2008) argues that customers prefer products and services of ESG companies from the perspective of shareholders and that corporate members can increase productivity through conducting CSR activities within the company. Jo and Na (2012) found that a firm’s active ESG activities reduce its overall risk, which drove a recent study by Díaz et al. (2021) showing that companies with high ESG were better able to withstand and overcome the crisis caused by COVID-19.

Because the concept of ESG is subjective and changes slightly over time, research on how to evaluate it has attracted attention. However, owing to the diversity of stakeholders, various methods for measuring corporate ESG performance have been proposed. Moreover, ESG is primarily concerned with managing the relationship between a company and its stakeholders such as consumers and communities. Therefore, according to Brammer et al. (2007) and Lee et al. (2016), ESG performance can be measured differently, depending on stakeholders’ interests.

Further, the diversity of stakeholders’ interest in ESG has created various institutions and measurement methods that evaluate ESG. Therefore, comparing and tracking changes in the evaluation of ESG has become an academic topic. For example, Escrig-Olmedo et al. (2019) analyzed how standards used by ESG institutions have changed over the past decade. Over time, standards have been consolidated in terms of environment and governance, rather than “sustainability.”

Most ESG business studies focus on uncovering the relationship between ESG (CSR) and a company’s financial performance. This is possible because many standards and evaluations generated by ESG rating agencies and the financial reports of numerous listed companies are readily available to researchers.

However, research on the ESG concept is relatively scant. The ESG concept is clearly explained using three keywords: environment, society, and governance. However, it is important to note that ESG is also a socially constructed concept, and the composition of that concept may vary with the influence of time and environmental changes.

### Topic Modeling and Sentiment Analysis in Business Research

Since their inception by Blei et al. (2003), topic models have become one of the most important fields of modern machine learning in natural language processing (NLP). Topic modeling is gaining traction because it allows for the definition of potential topics drawing on large unstructured text data (Indulska et al., 2012; Jeyaraj and Zadeh, 2020; Xiang et al., 2022). Storopoli (2019) explained the advantages of topic modeling as follows: First, topic modeling can be used to identify important topics that humans cannot discern. Second, it can be used to evaluate large-scale social phenomena. Third, the results of statistical validation eliminate the need for researchers to manually code and interpret data. Consequently, topic modeling has been widely applied in various fields such as geography (Cristani et al., 2008), political science (Quinn et al., 2010), hospitality and tourism (Lee, 2021) and medical science (Tran et al., 2019), etc. However, its application in business research has been quite limited.

Latent Dirichlet Allocation (LDA) has been applied in various fields. Nam et al. (2017) used LDA to extract hidden topics from brand perceptions of user segments. Kaplan and Vakili (2015) used LDA to develop text-based measures of unique patent ideas. Chae and Park (2018) analyzed CSR-related texts on Twitter to examine how CSR-related topics are being delivered, determine the main topics, examine how these topics are interrelated, and how they change over time.

Some business studies use an extended LDA model or develop a model based on LDA, including correlated topic modeling (CTM), to identify correlations between topics (Blei and Lafferty, 2007), structural topic modeling (STM) for discovering topics and revealing latent topical structures simultaneously (Otsuka et al., 2021), and the author topic model (ATM) for incorporating authorship information into the LDA (Rosen-Zvi et al., 2012). To measure business proximity, Shi et al. (2016) developed LDA and proposed a new method. Our study contributes to the second stream of research by applying dynamic topic modeling (DTM) to the analysis of Twitter data (Blei and Lafferty, 2006). The advantage of the DTM analysis is that it can detect changes in the trend of a subject over a time series.

Recent studies have attempted to measure human sentiments and predict future emotions through textual data analysis. Although it is difficult to accurately measure an individual’s sentiment, the sentiment accumulated through verbal expressions can be measured if certain criteria are given. Simply put, it is a method of arranging various words corresponding to sentiments and measuring the degree of sentiment through factors such as detection frequency (Medhat et al., 2014; Koto and Adriani, 2015). Sentimental analysis has developed following the development of text analysis technology as well as the growth of online social media platforms, such as Twitter and Facebook (Zimbra et al., 2018). Companies and various social groups have recently paid close attention to the value of messages and opinions generated on social media, which is also an important driver of research development.

Business research using sentiment analysis is largely divided into two types, according to the research subject. The first research stream investigates and forecasts stock market movements using sentiment analysis of online news or social media related to the stock market. Das and Chen (2007) analyze the sentiment of investors toward Amazon in the stock discussion on Yahoo from 1998 to 2005, and show the relationship between investor sentiment and stock value. Bollen et al. (2011) classified the sentiment of Twitter text into six moods (calm, alert, sure, vital, kind, and happy) as well as positive and negative emotions, and forecasted the fluctuations of the stock market price. Similarly, Bing et al. (2014) proposed a stock price forecast model based on the sentiment analysis of social media text. Previous studies mainly investigated the effect of opinions on stock prices. However, Deng et al. (2018) find that stock prices are expanding into their effect on microblog sentiment.

The second research stream involves the analysis of product or service reviews using sentiment analysis. These studies on customer reviews initially focused on numerical analysis, such as rating rankings and the number of reviews (Chevalier and Mayzlin, 2006; Huang and Chen, 2006; Archak et al., 2011). For example, Lu et al. (2013) show a positive correlation between review volume and sales revenue. In addition to the numerical analysis of customer reviews, the content of customer reviews was analyzed by analyzing the text. Text analysis is mainly performed based on sentiment analysis. For example, Hu et al. (2014) calculated the sentiment index by integrating the title and content of Amazon reviews, and showed that the sentiment index of reviews has a more direct effect on sales than customer ratings. Marchand et al. (2017) found that the volume of customer reviews influences sales of up to 10 weeks after the launch of a product, and positive/negative sentiments from reviews influence sales of up to 6 weeks. However, the amount of microblogging affects sales only during the first week of the launch. Gräbner et al. (2012) proposed a methodology for constructing a domain-specific lexicon to classify customer reviews into good or bad sentiments.

Few studies have used text analysis as a methodology in ESG. This is because the research subject was limited to the data disclosed by the company. Early studies were aimed at deriving ESG-related words and examining trends. However, it was difficult to analyze a large amount of information owing to the limitations of early text analysis technology. Nielsen and Thomsen (2007) analyzed reports from six companies, and Castellanos et al. (2015) used reports from seven companies. Since the mid-2010s, the development of big data handling technologies, such as deep learning and machine learning, has made it possible to automate and analyze large amounts of data. For example, Te Liew et al. (2014) used the term frequency-inverse document frequency (TF-IDF) methodology to analyze more than 100 companies. Recently, studies have applied the LDA model: Ning et al. (2021) analyzed 680 company disclosure data, and Raghupathi et al. (2020) derived automated results (topics) through 1,737 more reports.

Environmental, social, and governance-related text analysis has expanded its research topics from traditional initial public offering (IPO) studies to larger and broader areas, such as Twitter and Internet news. Chae and Park (2018) derived CSR-related topics through topic modeling using over 1.2 million Twitter posts via Twitter API. Lee et al. (2016) extracted approximately 17,000 CSR corpora from newspaper articles and research papers, and generated a CSR vocabulary based on them.

The subject of the study has been expanded from corporate disclosure data to Twitter and news, which means that ESG research, which has been narrowly studied from a corporate perspective, has been expanded to include a wider range of ideas and opinions from society. Given ESG is not only a topic of interest to companies, but an area of interest to various stakeholders such as the government, investors, and consumers, it is an appropriate change to expand ESG research to various social media platforms.

Most sentiment analysis research in the business domain focuses on stock market forecasts and the customer reviews of products or services. ESG-related sentiment analysis using corporate disclosure data has many difficulties in deriving managerial implications through sentiment analysis owing to the lack of positive disclosure data. Recently, studies have been conducted using social media data to overcome the shortcomings of corporate disclosure data with too many positive messages. Ballestar et al. (2020) showed that it is possible to classify Twitter users into groups of positive and negative sentiments by using ESG-related tweet text. This is because of the nature of Twitter data, in which various stakeholders express various positive/negative sentiments, unlike corporate disclosure data, which are naturally full of positive messages.

As shown in Table 1, some text studies use machine learning techniques, and some recent studies have started to apply sentiment analysis. Unlike previous studies that applied text analysis or sentiment analysis, our study applies both text and sentiment analyses to explore insights into ESG among the public and understand public reactions toward ESG.

**TABLE 1:** Text analysis (ML) and sentimental analysis about ESG.

Data	Method		
	Text analysis		Sentiment analysis
	Manual	ML/DL (Automated)	
Corporate Report (CSR Report, Disclosure, Financial Report, company website)	Nielsen and Thomsen, 2007; Gao, 2011; Castellanos et al., 2015; Lock and Seele, 2016	Te Liew et al., 2014; Liu et al., 2017; Lee and Huang, 2020; Kiriu and Nozaki, 2020; Raghupathi et al., 2020; Raman et al., 2020; Lee et al., 2021; Ning et al., 2021	Mućko, 2021
Social Media (Twitter, News…)	–	Lee et al., 2016; Chae and Park, 2018; Borms et al., 2021, Our study	Ballestar et al., 2020, Our study

## Data and Methodology

### Data

We chose Twitter as a data source because not only is it one of the most popular social media platforms, but is also a microblogging platform which provides data on real-time communication and information sharing in text form. Using #ESG and keyword ESG, we collected tweet data through Academic Research Track, a Twitter API that offers an unlimited time service for ten million tokens per month. The use of the term ESG began to increase rapidly after the Paris Climate Agreement in December 2015. We further set the data collection period from January 1, 2016, to December 31, 2020. We discarded retweets during this period using the Twitter API filter. The number of original tweets collected using the Twitter API with #ESG and ESG keywords totaled 1,787,230.

**FIGURE 1:**
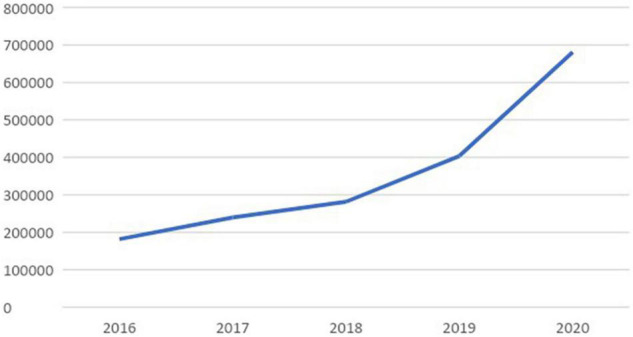
Number of tweets over time.

Figure 1 shows the volume of tweets collected by year. The number of ESG-related tweets continues to grow, reflecting the recent growing interest of businesses and the general public in ESG.

**FIGURE 2:**
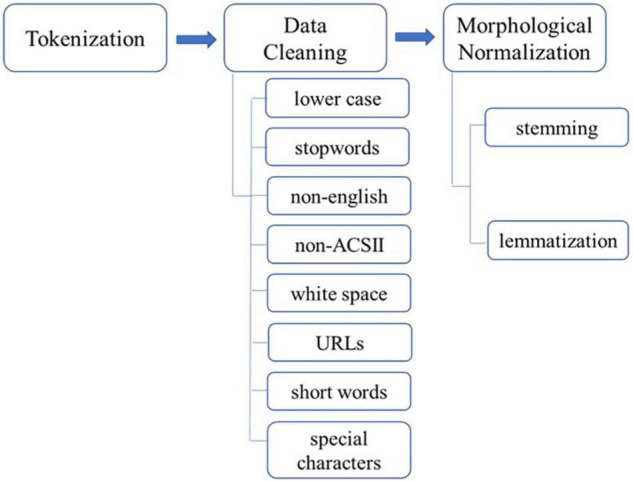
Diagram of data preprocessing.

The collected text data were then normalized, a process of transforming language that humans can understand into a standard form that can be understood by computers or machines. This normalization follows the procedures of tokenization, stop word removal, and morphological normalization. The diagram in Figure 2 shows the procedure of data preprocessing normalization.

First, tokenization was performed to break down each sentence and word into understandable minimum units. To smooth or remove noise in our textual data, we cleaned our data by converting all characters to lowercase, trimming non-English tweets, and eliminating stop words that are frequently used in the text but have no significant contribution to the meaning of the text, special characters, words less than four in length, unicode characters that are unreadable in an ASCII format, mentions, URLs, hashtags, and unnecessary white spaces. To remove stop words, we used the list of stop words provided by NLTK in Python. Third, stemming and lemmatization were performed for morphological normalization. Porter’s algorithm from the NLTK library was used to extract the stems of words. Using lemmatization, the inflectional endings of words are removed, and the root forms of words are obtained. Our preprocessing using Python resulted in 73,397,870 words.

For further empirical analysis, we converted our text data into a format that could be used as an input for the topic model. Each sentence in our dataset was sentenced into a list of words, after eliminating unnecessary characters such as emoticons and punctuation. The two main inputs to the topic model, corpus and dictionary, were further created.

The sentiment score for each tweet was measured using the Valence Aware Dictionary for Sentiment Reasoning (VADER), which is a lexicon and rule-based sentiment analysis available in Python. See section “Sentiment Analysis” provides more details on this measure.

### Model

#### Topic Model

**FIGURE 3:**
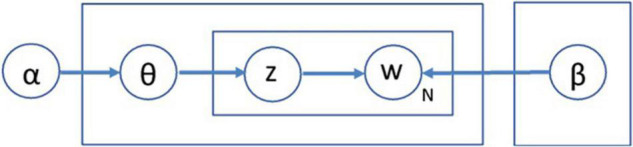
Plate notation representing LDA.

In summary, the topic model is an unsupervised machine learning model used to ascertain the latent thematic structure in a collection of documents with hierarchical probabilistic models. Among several algorithms such as latent semantic analysis (LSA), LDA has been one of the most widely used methods in topic modeling since its inception by Blei et al. (2003). LDA is a generative probabilistic model that describes a collection of documents as a text corpus. LDA presupposes a fixed number of topics, and further assumes that each document is represented as a distributional integration of these topics and words. As shown in Figure 3, the observed documents and words (w) in a dataset are generated using latent structures, such as the topic assignment of each word in each document (z), topic distribution of each document (θ), word distribution for each topic (β), and specific input number of topics, k.

The generative probabilistic process of document collection shown in Figure 3 can be summarized as follows:

1. For each topic *k* = 1, …, K,

generate β_k_ ∼ Dir (⋅| φ)

2. For each document *d* = 1, …, N,

generate θ_m_ ∼ Dir (⋅| α)

For each word w in document m,

generate z_m,n_ ∼ Mult (⋅| θ_d_)

generate w_m,n_ ∼ Mult (⋅| βz)

where Dir denotes a Dirichlet distribution and Mult denotes a multinomial distribution.

Using this hierarchical generative process of LDA, the joint distribution of the observed and hidden variables in Eq. 1 is obtained by:


(1) p⁢(β,θ,⁢z,⁢w)=⁢∏p⁢(β)⁢∏p⁢(θ)⁢∏p(z|θ)⁢p(w|β,⁢z)

The hidden structure of the observed and hidden variables was inferred by posterior inference using the Gibbs sampling MCMC algorithm.

Latent Dirichlet Allocation is very useful for performing the dimensionality reduction and qualitative summary of topics of large corpora; however, it does not consider the temporal order of when the tweets are written. We extended LDA into a dynamic topic model (DTM) by considering a topic as a sequence of distributions over fixed time intervals, such as years and months. Therefore, DTM allows us to discover the richer posterior structure of topics and explore the evolution of ESG-related topics over time and how the probability of top words within a topic changes over time. Considering the temporal aspect of documents is important because, for example, the term “ESG” did not even exist 15 years ago and has only been actively used recently.

**FIGURE 4:**
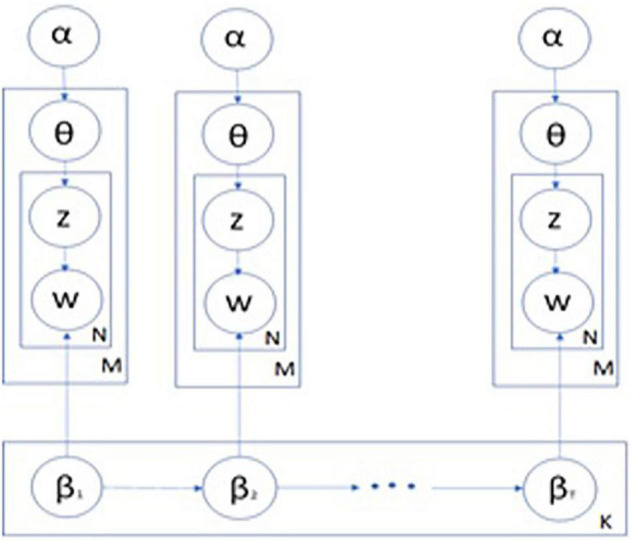
Plate notation representing DTA.

Dynamic topic model assumes that each document is arranged in a preset time span, each with its own topics. Each topic in each time span was randomly selected from the same topic in the previous time span. The probabilistic generative process of a document in a specific time span follows the same process as that of LDA. Figure 4 shows the plate notation of DTA, where *t* denotes the time slice, which is the year, and the other notations are the same as the LDA in Figure 3. Each topic at time *t* evolves from a corresponding topic at time *t-1*.

#### Sentiment Analysis

While topic modeling is an analysis of what topics are being discussed and how those topics evolve over time, sentiment analysis is a method used to understand the general public’s sentiments or opinions about a particular topic.

There are two major approaches to sentiment analysis: supervised machine learning, and unsupervised lexicon- and rule-based learning. This study was conducted using Python NLTK VADER (valence aware dictionary for sentiment reasoning (NLTK VADER) for the lexicon- and rule-based unsupervised learning methods). VADER is well suited for our study, as it is designed for sentiment analysis expressed on social media (Hutto and Gilbert, 2014).

Valence Aware Dictionary for Sentiment Reasoning provides a quantified sentiment output of compound scores for each sentence based on the polarity (positive or negative) and the intensity of the sentiment of each word. VADER uses a dictionary to map the lexical features of each word to its polarity and intensity, which are used to assess the sentiment of each word. By identifying the sentiment score of each word in a sentence, the compound score of sentiment in each sentence is calculated by summing the sentiment scores of each word in a sentence and then normalizing it to a number in the range of −1 to 1. A compound score greater than 0.5, less than −0.5 and between −0.5 and 0.5 is classified as positive, negative, and neutral, respectively. The number of tweets varies by date; for example, on October 29, 2020, there were 6,399 tweets, whereas on January 1, 2017, there were 159 tweets. We computed the mean compound scores for each date to determine the change in sentiment over time.

## Empirical Results

**FIGURE 5:**
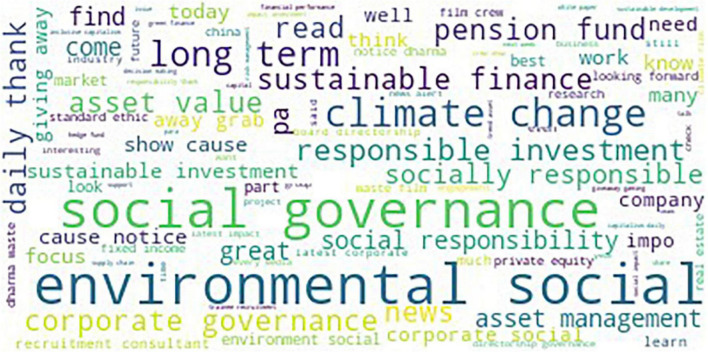
Word cloud of the most common cleaned words after normalization.

We generated the word clouds to visualize the important words based on their frequency, as shown in Figure 5. The word clouds in Figure 5 show the differences before and after normalization. Words that are not useful for text analysis, such as https and not RT in Figure 5, do not appear in Figure 5 after normalization.

**FIGURE 6:**
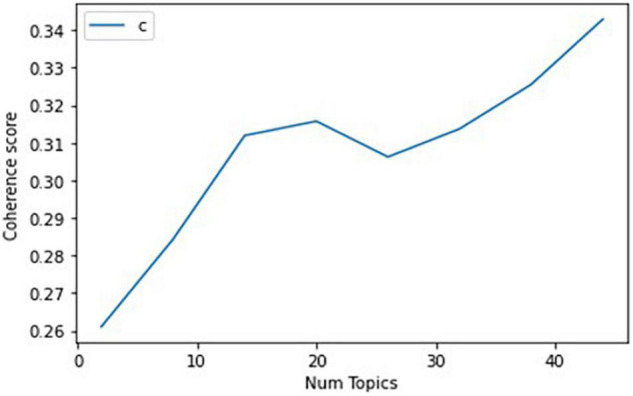
Coherence score for each number of topics.

To conduct LDA topic modeling, we used the Gensim-LDA library in Python. To derive meaningful results from topic modeling, it is important to determine the number of topics. This is because if the number of topics increases, various ESG-related trends can be extracted, but similar topics can occur, which can distort the evolution of each topic. In this study, 20 topics were chosen to produce the most interpretable and manageable number of topics, considering the tradeoff between semantic coherence in Figure 6 and exclusivity (Roberts et al., 2014).

Of the 20 topics found through topic modeling, we eliminated two topic clusters (topics 9 and 20) that were difficult or irrelevant to the label. The labels were chosen through discussion and consensus among the authors by reviewing the most likely tweets per topic and considering the top 30 most frequent words by topic and their probabilities. The 18 labeled topics and words that appeared the most in each topic are summarized in Table 2.

**TABLE 2:** Labeled topics and its top words.

Labeled topic	Best topic words	Number
Climate change	financial, trillion, billion, climate, change	1
Investment	investment, responsible, global	2
Asset value	value, asset, management	3
Fund	fund, pension, invest, credit	4
Carbon	carbon, reduce, impo	5
Stock	stock, exchange, ethical	6
Disclosure	disclosure, public, report	7
Business	business, responsibility, forward	8
Green	green, sustainable, energy, innovation	10
Data	data, analytics, performance, infrastructure	11
Investor	investor, executive, giveaway	12
Market growth	market, growth, performance	13
Conference	conference, join, panel, register	14
ESG	social, governance, environmental	15
Rating	rating, standard, socially, responsible	16
Training	trainee, guidance, forum, school	17
Transparency	transparency, trust, role, code	18
Consulting	consultant, research, money	19

**FIGURE 7:**
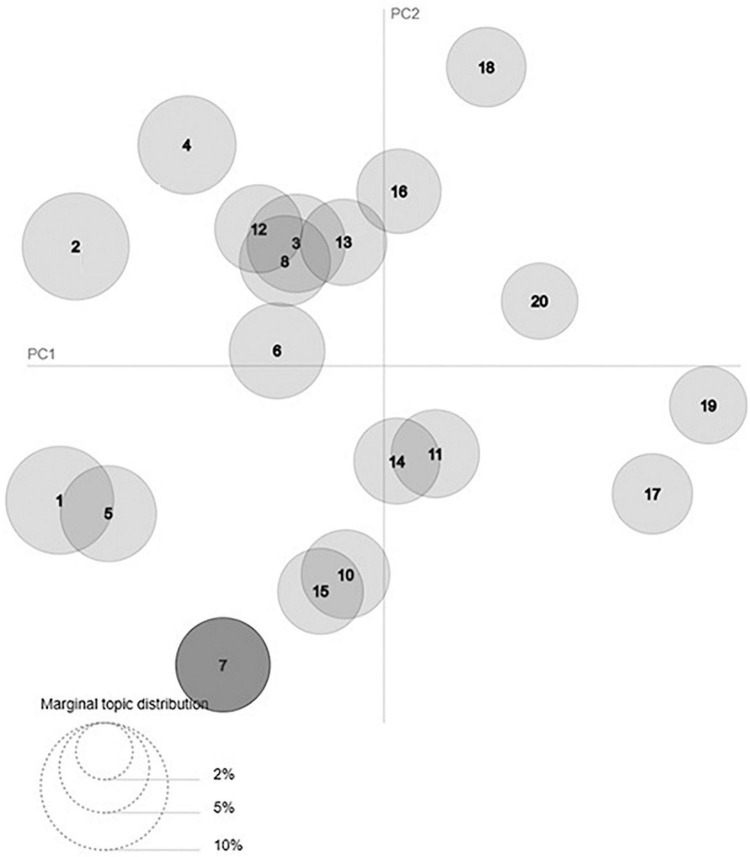
Visualization of intertopic distance map.

**FIGURE 8:**
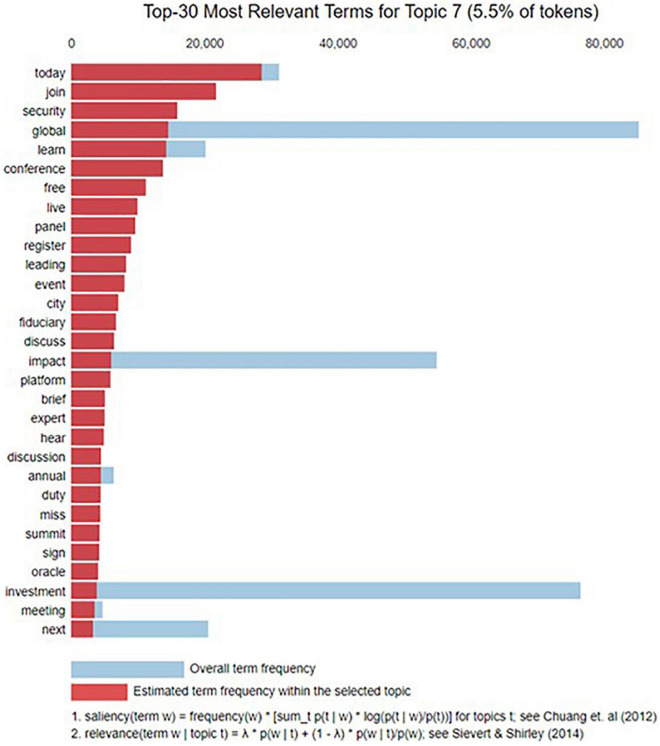
Bar chart of the top 20 most frequently occurring terms for topic disclosure.

Figures 7, 8 show a visualization of the topics in two dimensions. The visualization has two components: the intertopic distance map in Figure 7 and the bar charts in Figure 8. Figure 7 shows an overview of the topic model. Different topics are plotted as circles, where the importance of the incidence of each topic is indicated by the size of a circle. Using multidimensional scaling, the distance between each topic is expressed as the distance between the center points of each circle. For example, Figure 7 indicates that climate change in topic 1 with a relatively high share is far apart from transparency in topic 19, as climate change in topic 1 has some overlaps in words used with cluster 5, the topic of investor.

The 18 topics discovered and labeled in this study are climate change, investment, training, funds, investors, stocks, disclosure, data, carbon, conferences, business, market growth, green, ESG, rating, asset value, transparency, and consulting. Based on the intertopic distance map in Figure 7, the topic groups can be divided into four groups. The clusters of investment, fund, investor, asset value, business, stock, and market growth in the first quadrant of Figure 7 can be classified into investment groups. The climate change, carbon, ESG, and green clusters in the second quadrant of Figure 7 can be classified into environmental topic groups. Transparency and rating in the first quadrant and consulting, and training in the fourth quadrant of Figure 7 can be broadly classified into topics related to evaluation and education, respectively. In summary, ESG-related tweets were composed of 19 topics, which were further classified into four topics: environment, investment, education, and evaluation.

Figure 8 shows a bar chart in descending order of the top 20 most frequently occurring terms for topic disclosure. Overlaid bars indicate the corpus-wide and topical frequencies of a given term. We used the top 30 frequently used words to label each topic and interpret its meaning. Evidently, the terms “disclosure,” “report,” “risk,” and “financial” frequently appear in the cluster of topic 7.

Dynamic topic model results for topics of “climate change,” “green,” “rating,” “data,” “ESG,” and “investment” are shown in the following Table 3. For each topic, we selected the most interesting keywords and visualized them as time-series graphs to identify trends over time.

**TABLE 3:** Time series transition of probability of selected words in the topic.

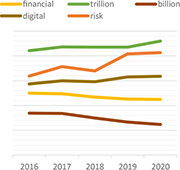	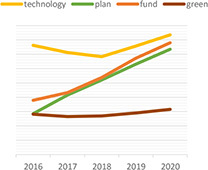
(A) Climate change	(B) Green

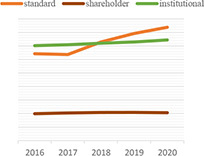	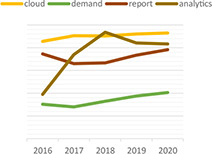
(C) Rating	(D) Data

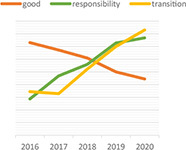	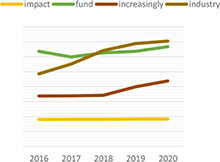
(E) ESG	(F) Investment

(A) “Climate Change” shows the annual trends for the probability of frequently occurring words – financial, trillion, billion, digital, and risk – within “Climate change” topic. The increasing use of the term “risk” in climate change topics seems to reflect growing awareness and interest in the risks associated with climate change. A decrease in the term “billion” and an increase in the term “trillion” among the unit words indirectly suggests that the risk of climate change is significantly increasing (Zhongming et al., 2021). The decline in the term “financial” and the increase in the term “digital” indicate that what previously focused on analyzing the risks of climate change financially is now starting to look for a solution, digital transformation.

(B) The term “green” shows that technology, plan, fund, and green are increasingly used within the “Green” topic. Given that this is a “green” topic to an environmental crisis, the growing use of these words translates into an effort to find alternatives in terms of the environment, one of ESG.

(C) The term “rating” shows that the use of the words shareholder, standard and institutional is a growing trend within the topic “Rating.” These trends reflect the growing importance of ESG evaluation standards and institutions performing evaluations in addition to shareholders’ interest in ESG.

(D) The term “data” shows that the demand for data analysis and reporting is increasing owing to the increase in ESG-related data.

(E) The abbreviation “ESG” shows that with respect to the ESG topic, the use of the words responsibility and transition is increasing and the use of the word good is decreasing. These trends indicate that the meaning of ESG is shifting from simply being a good company to one that is socially responsible.

(F) The term “investment” shows that the use of the words increasingly, industry, fund is increasing and the use of the word affect does not show a growing trend but it is consistently used. These trends indicate that interest in investing in ESG funds continues to grow, and the recent trend in which ESG funds are invested in by the industry sector is also recognized.

**FIGURE 9:**
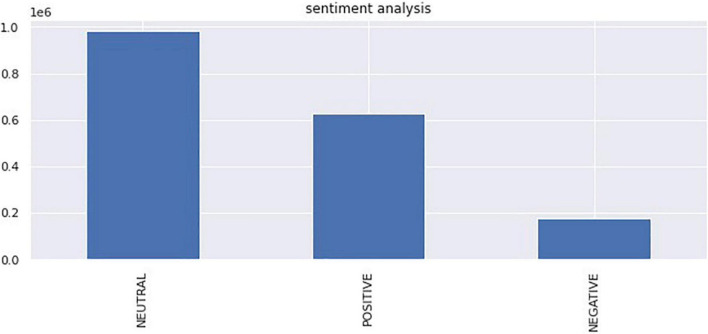
Bar chart of the sentiment score of each tweet.

**FIGURE 10:**
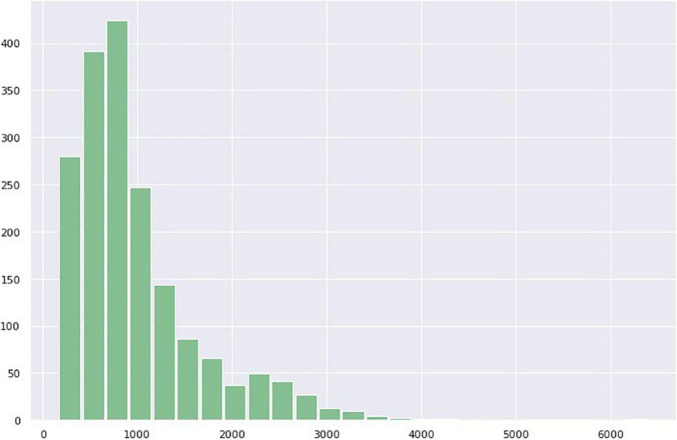
Histogram of the amount of twitter per day.

The number of tweets varies by date; for example, on October 29, 2020, there were 6,399 tweets, whereas on January 1, 2017, there were 159 tweets. The number of Twitter posts per day in Figure 9 shows that it follows a right-skewed distribution. Therefore, the sentiment score on a particular day was calculated by computing the mean of the compound scores for each date. Figure 10 shows a histogram of sentiments per day that appears to follow a normal distribution with a positive mean.

Figure 9 shows that more than approximately half of public sentiments are neutral and that positive sentiments are approximately three times more common than negative sentiments. These findings could be understood by noting that the ESG concept should be viewed from two perspectives. Fundamentally, it aims for positive values that pursue corporate responsibility in terms of environment, society, and governance, and simultaneously serves as a standard for evaluating the activities of companies pursuing these values. The former can be interpreted as positive sentiments and the latter as neutral sentiments.

As shown in Figure 10, the number of tweets per day follows the right skew distribution, and the tweet volume changes daily. We computed the mean compound scores for each date to determine the change in sentiment over time.

**FIGURE 11:**
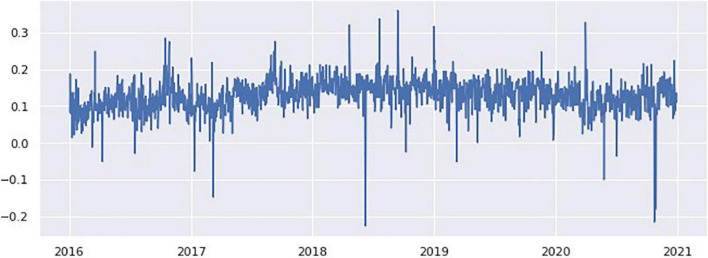
Public sentiment of tweets on ESG with timeline.

Figure 11 shows that the sentiment of public tweets for ESG was calculated for each date as the timeline fluctuates considerably depending on the date. The biggest peak occurred on September 14, 2018 with a sentiment score of 0.359, but was not accompanied by a surge in the amount of tweet volume. A closer look at the tweets on September 14, 2018 reveals why. One of the main reasons for this is that the global climate action summit (GCAS) was held for 3 days, from September 12, 2018 to September 14, 2018. Heads of state, government representatives, industry and civil society leaders, and officials from international organizations gathered to discuss specific plans for climate change, such as the implementation of the Paris agreement. On the 14th, the last day of the summit, positive tweets about ESG activities seem to have exploded owing to the activities of GCAS.

April 22, 2018, was also one of the days when positive sentiment surged. A closer look at the tweets indicates that it was designated as Earth Day to show support for environmental protection. Earth Hour, which advocates switching off lights at 8:30 pm in more than 193 countries, is a very popular Earth Day event. Moreover, the success of these light-out events has been instrumental in the surge in positive ESG tweets.

The significant increase in negative sentiment tweets on October 24, 2018 (score −0.216) and October 29, 2018 (score −0.180) appears to be related to President Trump’s conservative stance on environmental policy. During the United States presidential debate between Trump and Biden on the 23rd, Trump made headlines online when he made anti-environmental remarks, “wind energy is very expensive. kills all the birds.” On the 29th, an article was published stating that a bill to ban retirement pensions from investing in ESG funds was being pursued quickly, which drew criticism from many ESG investors.

Regarding the surge in negative sentiment on October 24, 2018, negative newspaper article feedback appeared to have an impact on ESG. A rare negative comment on ESG in the *Financial Times*, “The fallacy of ESG investing: Win-win arguments promoting both bigger profits and better social returns are illogical” seemed to trigger many negative tweets.

The reason for the high negative sentiment score on June 9, 2018, is interesting. This is because of the surge in negative sentiments caused by tweets about the losses incurred by an online gaming team that uses the abbreviation ESG. For further research, tweets about the game team ESG should be eliminated as noise. See Table 4 for examples of tweets from days when positive and negative sentiments surged.

**TABLE 4:** Examples of tweets from days when positive and negative sentiment surge.

Date	Tweets
14-09-2018	@DanielRossPerry “You have to face this now. You’re going to feel pressure from investors, employees, consumers, vendors. It’s a fabulous opportunity for entrepreneur. Don’t work on your #ESG story, figure out your #ESG impacts” inspiring stuff to wrap up @NasdaqTech #GCAS2018
14-09-2018	@evanharvey99 “When should #starups thinking about #ESG? Right now” @Nasdaq @nasdaqcenter @GCAS2018
22-04-2018	@BradZarnett We must act before it’s too late. On Earth Day today commit to the planet! Thanks Nath Paresh for this powerful cartoon! #EarthDay #EarthDay2018 #ESG #climate #climate change
22-04-2018	@kmahnhw Happy #EarthDay! Good time to look at #sustainable, responsible and #impact investing with @SmartTrustUIT #ESG
24-10-2020	@ChrisLu44 Trump literally doesn’t understand anything about wind or solar power.
24-10-2020	@TomPowdrill This article seems to have annoyed a lot of people in my microcosm, but I think it has a lot of sense in it. “The fallacy of ESG investing”
24-10-2020	@Benefits_PRO Trump administration moves at “warp speed” to kill ESG in retirement plans
09-06-2018	@Sky_LoL_At How can a pta fiora, who shittalks entire game, perma ff, starts dorans blade vs. pantheon, keeps 1v1 after being 0 3, get into ESG. Idk man, this guy every single time I see him he talks so much SHIT

Although we presumed and investigated that there could be a correlation between the number of tweets and sentiment score in ESG, an insignificant Pearson correlation between tweet volume and sentiment score (*r* = 0.110) indicates that conversation volume is not significantly related to the sentiment score.

The above results show how text analysis using LDA, DTM, and sentiment analysis can help uncover narratives in unstructured text data.

## Discussion

Authors’ conversations in social media change rapidly so that we can determine the general public’s reaction or emotion to a specific topic at this point in time. ESG is widely used to evaluate non-financial factors such as corporate sustainability and social responsibility, and its importance is being increasingly emphasized. This study proposes a method to obtain real-time information and track evolution on the topics the general public is talking about ESG and how the public feels about it using publicly available Twitter data.

By applying LDA, DTM, and sentiment analysis, we were able to identify unobserved topics and sentiments in a collection of ESG-related Twitter posts and ascertain how they evolve over time. DTM was performed on seven out of 18 topics found through LDA, which allowed us to reveal characteristic evolutionary processes by time-series tracking of seven topics and frequently used words within each topic.

Our study shows that ESG-related tweets are composed of 18 topics, which are further classified into four groups: environment, investment, education, and evaluation. Among these, the most frequently mentioned topic is investment. In Figure 7, seven topics (investment, asset value, fund, stock, investor, business, and market growth) out of a total of 18 topics are located close to each other in the investment group, whereas in the ESG cluster closest to a pure ESG concept, there are only four labeled topics (environment, carbon, green, ESG). There are two topics near the investment group: rating and transparency, which can be considered extensions of ESG investment from an ESG evaluation point of view.

The background of ESG growth means that there is a need for investors to evaluate and invest in the value of a company’s sustainable growth. Corporate CSR activities have been implemented to enhance social pressure and corporate image, and whether these activities actually increase corporate profits is controversial in academia and management fields; however, investors are not interested. In a situation in which the capitalist system and the sustainability of the planet are at stake, such as the global financial crisis and global warming problem, and COVID-19, investors are also aware of the crisis and are encouraging and pressing companies to solve ESG issues. The results of our study explain this social phenomenon.

We show that identifying the evolution of frequently used words in each topic over time could provide an opportunity to gain a broader understanding of the analysis and predictions about current and future trends in that topic. For example, the increasing use of the word “risk” in climate change topics reflects growing awareness and interest in the risks associated with climate change. Additionally, words such as, “technology,” “plan,” and “fund” are increasing in green topics, implying that resources such as alternative green technologies and investment to address current environmental challenges are increasing, and these investments are expected to continue in the future.

We show that daily sentiment scores fluctuate over time without any particular pattern, and that most sentiment scores are positive when summed over days. For specific days with significantly higher or lower sentiment scores, it is most understandable if we consider the tweets on that day. For example, sentiment in the September 14, 2018 tweets appear to have surged to high positive values on the final day of the global climate action summit (GCAS) as a result of the 3-day GCAS activity.

In this study, we applied LDA, DTM, and sentiment analysis to derive potential topics related to ESG and ascertain how the ESG theme is structured and changes over time and how the public’s emotions are related to this change. Whereas existing text analysis studies on ESG mainly apply simple text analysis methods or topic modeling methods, our study is meaningful in that we take an integrated approach to ESG themes by adding time-series analysis (DTM) and sentiment analysis.

The results of this study are managerially and practically relevant. The results of our research can serve as a guideline for companies or organizations with a poor understanding of ESG, to easily prepare for ESG management. For example, ESG is a relatively recent concept that has received a lot of attention since the Paris Climate Agreement. For companies that understand ESG as an extension of CSR unrelated to investment or simply as being a good company, our research helps clarify that ESG is a new concept linked to investment. The sentiment analysis of ESG fluctuates and has no specific pattern. It can be used as an appropriate media response tool by analyzing ESG in real time for a specific company or organization.

## Limitations and Future Research

This study has some limitations. LDA and DTM are general topic models. However, they are based on simplified assumptions. First, models are required for the number of topics to be predetermined and fixed in advance, which is subjective and may not reflect the true distribution of topics. Second, the models are based on the bag-of-words assumption that the order of words in a document and that of documents can be ignored. Future research should consider using extended topic models that consider word dependency and are more flexible in their basic assumptions, such as the extended global topic random field (Wang et al., 2015).

In most previous research on topic modeling, the topics in each cluster were manually named, and in this study, the topics were manually named according to previous studies. However, this method may result in judgment bias that is influenced by the subjectivity of the researcher. Therefore, in future studies should strive to minimize judgment bias through the automatic naming of word clusters without human intervention, as in Hindle et al. (2013).

Twitter is the most popular social media platform for academic research because it makes it easy to obtain data through an application programming interface (API). However, tweet data contain many expressions that differ from everyday life, such as slang, emoticons, hashtags, and ironic sentences, which makes it difficult to find the exact meaning using text-mining techniques. These difficulties pose problems for many natural language processing methods such as sentiment analysis. Because results can vary significantly by using different types of stemmers or lemmatizes and extraction of data, it is important to try various and tuned methods using a wider range of social media text data.

A major challenge in using social media data to identify real-world trends is the bias caused by the self-reporting nature of social media. As discussed in organizational behavior research (Podsakoff et al., 2003) a common technique for mitigating self-reporting bias is collecting self-reported data through experience sampling methods; this is not applicable to a passive setting of social media analysis. Recently, Sheth (2009), Cheng et al. (2011), and Kıcıman (2012) showed the need for a better understanding of the self-reporting bias inherent in social media by integrating social media analysis with location-based social networks. The problem of self-reporting bias inherent in social media data is considered an important future research stream because little empirical research has been conducted relative to its importance.

To deepen our understanding of the dynamics of topics and sentiments about ESG, future research could conduct analyses that consider the dynamics and evolution of topics and sentiments together. In this study, topic modeling and sentiment analysis were conducted separately. Because we can conduct sentiment analysis using the probability of words that are frequently used in each topic, it is important to understand the complex dynamics if we understand how evolution over time of topics is related to the sentiment score and how it is affected by sentiments. Second, many ESG-related studies suggest a positive effect of ESG activities, including financial performance, on companies. Collecting ESG-related tweets about individual companies and analyzing relationships between sentiments and financial performance, such as stock price, are expected to contribute to existing research. Third, many studies have found that companies with high ESG are strong against risk. Computing the ESG scores of individual companies using tweet data and examining the role ESG plays in the crisis triggered by COVID-19 would be interesting. An event study could be used to observe stock price changes before and after the outbreak of COVID-19.

## Data Availability Statement

The raw data supporting the conclusions of this article will be made available by the authors, without undue reservation.

## Author Contributions

S-UJ: study conception and design. WC: data collection. S-UJ and JP: analysis and interpretation of results and draft manuscript preparation. All authors reviewed the results and approved the final version of the manuscript.

## Conflict of Interest

The authors declare that the research was conducted in the absence of any commercial or financial relationships that could be construed as a potential conflict of interest.

## Publisher’s Note

All claims expressed in this article are solely those of the authors and do not necessarily represent those of their affiliated organizations, or those of the publisher, the editors and the reviewers. Any product that may be evaluated in this article, or claim that may be made by its manufacturer, is not guaranteed or endorsed by the publisher.
